# Exploiting Bioprocessing Fluctuations to Elicit the Mechanistics of *De Novo* Lipogenesis in *Yarrowia lipolytica*

**DOI:** 10.1371/journal.pone.0168889

**Published:** 2017-01-04

**Authors:** Andreas E. Vasdekis, Andrew M. Silverman, Gregory Stephanopoulos

**Affiliations:** 1 Department of Physics, University of Idaho, Moscow, ID, United States of America; 2 Environmental and Molecular Sciences Laboratory, Pacific Northwest National Laboratory, Richland, WA, United States of America; 3 Department of Chemical Engineering, Massachusetts Institute of Technology, Cambridge, MA, United States of America; INRA, FRANCE

## Abstract

Despite substantial achievements in elucidating the metabolic pathways of lipogenesis, a mechanistic representation of lipid accumulation and degradation has not been fully attained to-date. Recent evidence suggests that lipid accumulation can occur through increases of either the cytosolic copy-number of lipid droplets (LDs), or the LDs size. However, the prevailing phenotype, or how such mechanisms pertain to lipid degradation remain poorly understood. To address this shortcoming, we employed the–recently discovered–innate bioprocessing fluctuations in *Yarrowia lipolytica*, and performed single-cell fluctuation analysis using optical microscopy and microfluidics that generate a quasi-time invariant microenvironment. We report that lipid accumulation at early stationary phase in rich medium is substantially more likely to occur through variations in the LDs copy-number, rather than the LDs size. Critically, these mechanistics are also preserved during lipid degradation, as well as upon exposure to a protein translation inhibitor. The latter condition additionally induced a lipid accumulation phase, accompanied by the downregulation of lipid catabolism. Our results enable an in-depth mechanistic understanding of lipid biogenesis, and expand longitudinal single-cell fluctuation analyses from gene regulation to metabolism.

## Introduction

Lipid droplets (LDs) are cytoplasmic emulsions, capable of storing neutral lipids such as triacylglycerols (TAG) and steryl esters (SE) at varying ratios [1–4]. By undergoing enzymatic hydrolysis, these stored compounds serve several cellular needs, such as membrane and lipoprotein biogenesis, as well as provide precursors towards oxidation mediated energy production. Similarly, autophagy may degrade LDs (i.e. *lipophagy*), whereas the LD content is directly released into lysosomes for further degradation [5]. LDs have attracted significant excitement in recent years as cost-effective biodiesel precursors in renewable energy production [6]. To this end, *Yarrowia lipolytica* has emerged as a model oleaginous yeast due to its genetic tractability, as well as enhanced lipid accumulation capability–most in the form of TAG [7–9]. In addition to industrial applications, more active roles of LDs have been recently recognized, such as their interactions with other organelles to coordinate immune responses [10], as well as cell protection against lipotoxicity [11].

Different pathways may induce lipid accumulation [12]. These include: (1) direct fatty acid internalization, esterification and incorporation to LDs [11]; and (2) *de novo* fatty acid synthesis through the mitochondrial TCA cycle and Kennedy pathway utilizing carbon precursors such as glucose and acetate [13]. According to the current consensus, the endoplasmic reticulum (ER) is the origin of LDs in most single-cell organisms [3, 4, 14]. This view is primarily based on the observation that essential enzymes to lipid biosynthesis reside in the ER [15], including diacylglycerol acyltransferase (e.g. DGAT1)–an enzyme involved in the final step in TAG biosynthesis. This LD biogenesis mechanism suggests that cytosolic lipid accumulation occurs primarily through the increase of the number of cytosolic LDs. More recently, an alternative mechanism of lipid accumulation was reported, evidencing that cytosolic LDs can also grow by size [16]. To this end, the glycerol-3-phospate acyltransferase (GPAT4), as well as diacylglycerol acyltransferase (DGAT2) were identified as essential components of those LDs that grow by size. Interestingly, the GPAT4 isoenzyme was not found to decorate all cytosolic LDs, but rather a smaller portion of them. This enzyme localization heterogeneity was identified as a mechanism generating two diverse LD populations: those that grow in size, and those remaining “static” [16].

**Fig 1:**
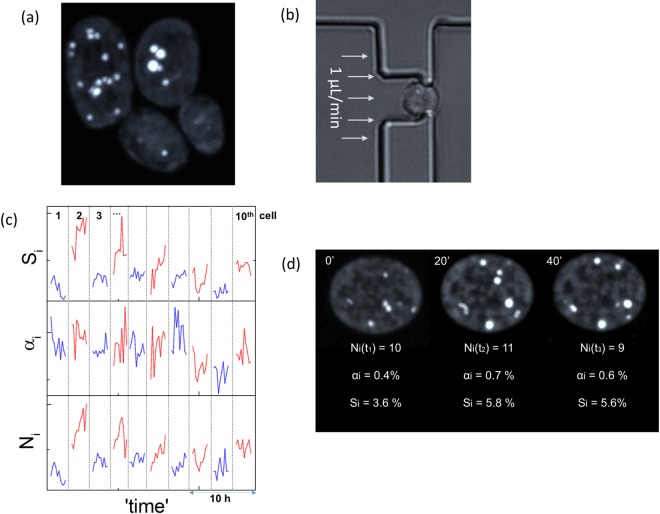
(a) A maximum intensity projection of two budding *Y*. *lipolytica* Po1g cells, indicating two lipid-content phenotypes, namely: *large-but-fewer* LDs and *small-but-more* LDs. (b) A single Po1g cell trapped in the microfluidic system under continuous laminar flow at 1 μL/min. (c) Single-cell longitudinal time traces of the number of cytosolic LDs (N_i_), their median area (α_i_) and the percentage of lipid content (S_i_); 10 single-cell traces are illustrated, in varying colors consecutively. (d) Time-lapse 3D imaging of neutral lipid fluctuations (N_i_, α_i_, S_i_) of a single Po1g cell.

Another lipogenesis aspect that has also attracted substantial attention in recent years is the persistent cell-to-cell lipid content heterogeneity. A recent report identified this form of heterogeneity as a non-heritable trait, as well as its protection role against lipotoxicity [11]. To a similar end, we observed at the single-cell level that cytosolic lipid accumulation is far from monotonic with time [17]. We identified this form of bioprocessing noise as the origin of the cell-to-cell heterogeneity, confirmed its epigenetic origins and dependence on the extracellular environment [17]. In addition to the cell-to-cell lipid content heterogeneity, another form of phenotypic heterogeneity persists in clonal populations, whereas some cells contain *large-but-fewer* LDs, and others contain *small-but-more* LDs. A representative example of this innate phenotypic bistability is illustrated in Fig 1A for the Po1g strain of *Yarrowia lipolytica* [7–9]. While this form of phenotypic bistability has been appreciated since the first electron micrographs of yeast (see for example: [18]), they have yet to be extensively examined.

Despite substantial recent progress in identifying the different biochemical pathways of lipid accumulation [19], including the transcriptional regulatory changes under nitrogen starvation [20, 21], observations similar to those of Fig 1A still question the mechanistics of lipogenesis, namely: does lipid accumulation occurs primarily through the number of cytosolic LDs, or through their size? To answer this question, we explored neutral lipid expression at the single-cell level using microfluidics and optical microscopy. The approach was inspired by the plethora of single-cell analyses that have elucidated many features of gene regulation, which are otherwise hidden in bulk–population level–assays [22, 23]. Rather than gene expression, we probed the lipid content in *Y*. *lipolytica*, a model oleaginous organism [7–9, 13, 21, 24, 25]. Specifically, we selected the Po1g strain, which is transformed to overexpress the acetyl-CoA carboxylase (ACC) and diacylglycerol transferase (DGAT) genes, thereby allowing it to accumulate lipids [8]. By placing this strain within a microfluidic quasi-time invariant microenvironment–a crucial parameter in longitudinal analyses given the highly dynamic nature of the metabolome [26]–we previously reported the sporadic nature of lipid accumulation at early stationary phase [17]. Here, we employ these incessant fluctuations to statistically correlate the two phenotypes pertaining to *large-but-few* versus *small-but-many* LDs, and investigate the mechanistics of lipid accumulation and degradation under rich medium and protein translation inhibition steady-state conditions.

## Results

### Visualizing regulatory mechanistics

Following our previous analysis, the intracellular neutral lipid content (**S**_**i**_, namely total LD area normalized over the cell area) was observed to fluctuate strongly over time [17]. The S_i_ fluctuations were manifested both through fluctuations of the number of LDs (**N**_**i**_), as well as their average sizes (**α**_**i**_**−**normalized over the cell size)–illustrated the longitudinal traces for 10 *Y*. *lipolytica* Po1g cells in Fig 1C, as well as in the single-cell images of Fig 1D. To attain a deeper insight into both the synthesis and degradation of neutral lipids, we derived the instantaneous lipid flux in accordance to the equation:

$$\frac{dS_{i}}{dt}=\alpha_{i}\frac{dN_{i}}{dt}+N_{i}\frac{d\alpha_{i}}{dt}$$ (1)

**Fig 2:**
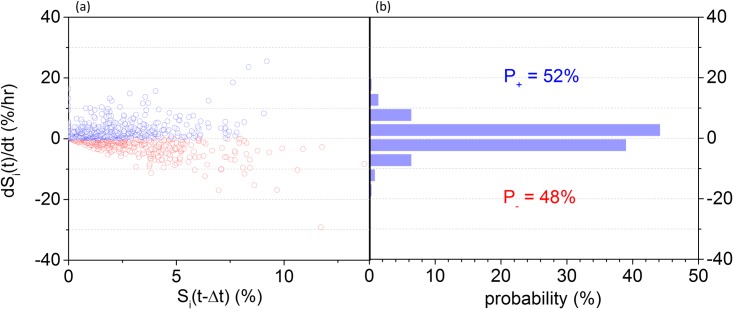
(a) A scatter plot of the lipid flux (dS_i_(t)/dt) as a function of the total lipid content (S_i_(t-Δt)); each point represents the instantaneous flux per cell, with positive ones plotted in blue, and negative in red. (b) A probability histogram of the lipid flux for all observations (n = 80 cells).

The instantaneous lipid flux can be either positive or negative, pertaining to a higher probability of lipid accumulation rather than degradation within the observation window (Δt). The latter was chosen to be Δt = 20 min (Fig 2A), limited by the sampling duration during 3D imaging, and the number of single-cells per chip. For Po1g, the positive and negative median fluxes were similar (within 6%), at 2.8%/hr (Fig 2B). This indicates that both lipid accumulation and degradation in *Y*. *lipolytica* occur at rates of comparable magnitudes. Despite their similar magnitudes, the positive lipid-flux observations during the 140 min of immobilization were marginally more likely (52% probability in total) than the negative ones (48% in total) as plotted in Fig 2B. This probability distribution indicates a moderately higher probability of *de novo* lipogenesis over degradation within the 140 min observation window.

Additionally, the lipid flux (dS_i_/dt) is weakly correlated to its content (S_i_) at any instant (Fig 2A), with a Spearman correlation coefficient *ρ = 0*.*23* (P = .001). To explain this, we developed a coarse-grain (deterministic) model, whereas the lipid flux depends on the activity levels and concentration of associated enzymes, as well as the substrate concentrations [27, 28]. This can be qualitatively described as follows (see S1 Document for a full derivation) [29]:

$$\frac{{dS}_{i}\left(t\right)}{dt}=\frac{\kappa^{+}\left(t\right)∙E^{+}\left(t\right)}{dt}∙C_{i}+\frac{{\kappa^{+}\left(t\right)∙E^{+}\left(t\right)+\kappa }^{-}\left(t\right)∙E^{-}\left(t\right)}{dt}∙S_{i}\left(t-\Delta t\right)$$ (2),

where κ^+^ and κ^-^ are the reaction rates of the enzyme manifolds associated with lipid accumulation (E^+^) and degradation (E^-^), and C_i_ is the available cytosolic carbon substrate dedicated to fatty acid synthesis and elongation.

Under the assumption that C_i_ is at equilibrium with the time-invariant extracellular supply, the weak S_i_−dS_i_/dt correlation suggests that the rate of *de novo* lipogenesis is primarily bounded by the rates of enzyme concentrations and activities and less by the intracellular substrate and product levels (Eq 2). Alternatively, the rate limiting step in expressing or degrading lipids rests with the enzyme reaction rates, rather than the cytosolic substrate or product concentrations. This conclusion is in agreement with our previous finding that bioprocessing noise depends inversely on the intracellular lipid content: in essence, the bounded lipid flux (dS_i_/dt) enforces stronger (weaker) lipid content (S_i_) fluctuations at low (high) cytosolic product concentrations [17].

### LD copy-number or size?

**Fig 3:**
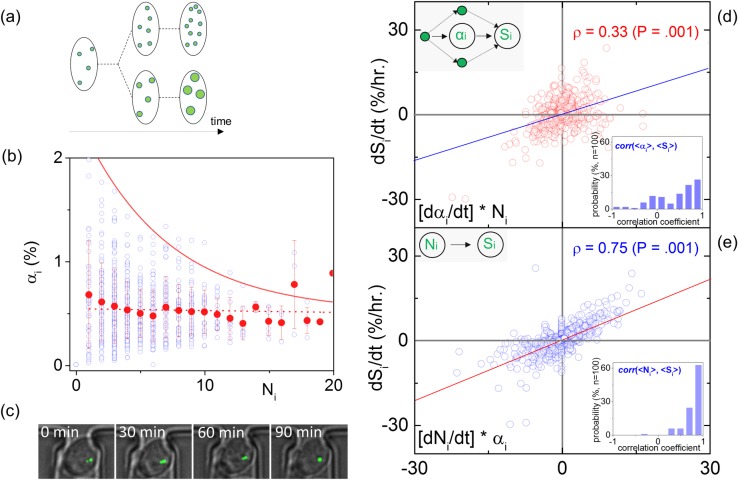
(a) A schematic illustration of the two possible mechanistic pathways for lipid accumulation, namely by accumulating more LDs or larger LDs. (b) The dependence of N_i_ on α_i_ (blue dots), including the mean values of α_i_ per N_i_ (red dots–error bars denote the standard deviation), as well as the exponential decay fit of the α_i_ maxima for increasing N_i_ (red solid line). (c) The dependence the lipid flux (dS_i_/dt) on [α_i_ ∙ dN_i_/dt]; red circles denote observations per unit time and the solid blue line is the linear fit; *upper inset* depicts the cause-effect relationship between S_i_ and α_i_; *lower inset* plots the histogram of the [αi—S_i_] correlation coefficient per single-cell. (d) Similar to (c), the dependence of dS_i_/dt on [N_i_ ∙ dα_i_/dt] is plotted, including the observations (blue circles), a linear fit (solid red line), the causality diagram (upper inset) and correlation coefficient per cell histogram during the observation window.

To investigate whether lipid accumulation occurs via the copy-number of cytosolic LDs versus the LDs size (Fig 3A) in the WT *Y*. *lipolytica*, we probed the relationship between the LDs copy-number with the median LD size–per cell at all instances. The scatter-plot of Fig 3B illustrates the inter-dependence between these variables for approximately *n = 80 cells*. It can be observed that N_i_ varies–as expected–in integer steps, while the α_i_ values fluctuate for each N_i_. Despite such fluctuations however, the median α_i_ value remains relatively constant for all N_i_. An exception to this apparent inter-dependence are the decreasing maximum values of α_i_ for increasing N_i_ (solid red line–Fig 3B). This indicates while variable size LDs can be produced, their maximum size is ultimately bounded by the number of LDs. Namely, while *Y*. *lipolytica* cells can produce variable size LDs, their maximum size is ultimately limited by the number of intracellular LDs.

The weak correlation between the number of LDs (N_i_) and their area (α_i_) for all cells at any given instance observed in Fig 3B questions which of the two variables is most integral to lipogenesis, and lipolysis. Does lipid accumulation occur through N_i_ in accordance to the consensus that LDs originate from the ER [4], or through α_i_ in the presence of essential isoenzymes (Fig 3A) [16]? Additionally, is LD degradation a digital process through N_i_, or can degradation occur through analogue decreases in α_i_?

To investigate the causality between α_i_ and N_i_ with S_i_, we plotted the lipid flux (dS_i_/dt) as a function of its two weighted components, namely [N_i_ ∙ dα_i_/dt] and [α_i_ ∙ dN_i_/dt] (see Eq 1), shown in Fig 3C and 3D. The lipid flux relationship with the rate of LD size change is complex and weakly correlated (Fig 3C) with a Spearman correlation coefficient of *ρ = 0*.*33* (P = .001). This weak correlation suggests the presence of compensating mechanisms (see inset’s pathway diagram). Such compensating mechanisms dephase the S_i_ - α_i_ relationship, thereby limiting its causality [23]. This behavior is noted for both positive and negative instantaneous lipid fluxes (dS_i_/dt).

On the contrary, increased linearity between dS_i_/dt and dN_i_/dt is observed with a *ρ = 0*.*75* Spearman correlation coefficient (P = .001) as illustrated in Fig 3D. This occurs for both positive and negative fluxes, indicating the causal relationship between S_i_ and N_i_ (see inset in Fig 3D). The higher linearity between dS_i_/dt and dN_i_/dt (Fig 3C) than dS_i_/dt and dα_i_/dt (Fig 3D) manifests that intracellular lipid accumulation and degradation are substantially more likely to occur through fluctuations in the number of LDs (N_i_) rather than their average size (α_i_). The outcome of the preceding instantaneous lipid flux analysis is in agreement with the [N_i_—S_i_] and [α_i_—S_i_] correlations per single-cell time series, as shown by the inset histograms of Fig 3C and 3D.

### Protein translation and lipogenesis

**Fig 4:**
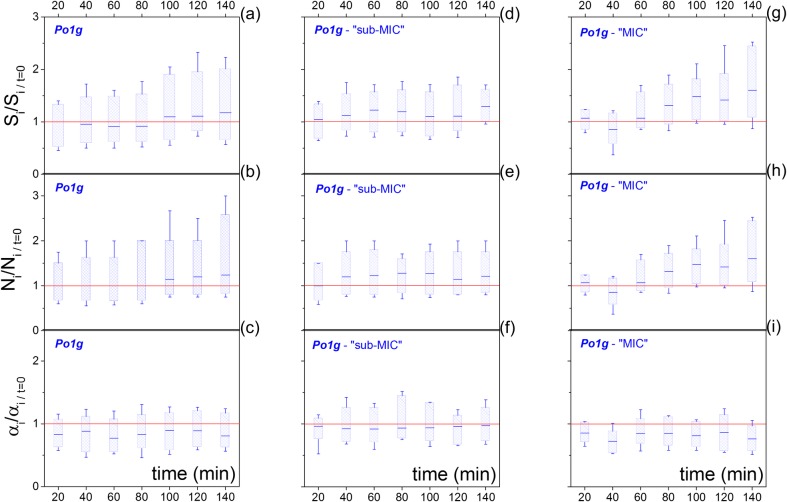
(a-c) Boxcharts of the longitudinal fluctuations of the lipid content (S_i_−a), number of LDs (N_i_−b) and median LD size (α_i_−c). The boxcharts represent the median, 25% - 75% range, and the whiskers the 20% - 80% range. All single-cell observations are normalized to initial value at t = 0 for each cell-lineage. (d-f) and (g-i) illustrate the same longitudinal traces for the sub-MIC (d-f) and for the MIC (g-i) concentration of CHX.

Our final investigation focused on lipogenesis under protein translation inhibition conditions. Under continuous (time-invariant) exposure to rich medium, the Po1g cells did not exhibit considerable neutral lipid increase during the microfluidic immobilization (140 min). This is illustrated in Fig 4A, where the lipid content (S_i_) is plotted as a box-chart time series, where each box-chart is normalized at the S_i_ value of t = 0 for each individual cell lineage. During the first 100 minutes in YPD medium, on average lipid degradation took place (i.e. S_i/normalized_ < 1 for 0min < t < 100min), followed by a 17% lipid increase at 140 min. This increase is probabilistic, and reflects the median response of the population, whereas 39% of the population exhibited lipid degradation, rather than accumulation. In agreement with the preceding analysis, lipid accumulation S_i_ takes place primarily through the number of cytosolic LDs (N_i_), rather than their area (α_i_). This is illustrated in the time dependent box-charts of Fig 4B and 4C, whereas–unlike α_i_−the median N_i_ is monotonic with S_i_.

Subsequently, we introduced cycloheximide (CHX) in the YPD medium flowing in the microfluidics. CHX acts on the E-site of the 60S ribosomal subunit, thus blocking the translocation step during elongation [30]. As a result, CHX inhibits protein translation, thereby suppressing growth and increasing the cytosolic concentration of free amino acids, which has been previously linked to the enhanced activity of the mTORC1 (Mammalian Target of Rapamycin) complex [31]. Therefore, by introducing CHX in the medium, protein translation and cell growth were artificially suppressed. Such ‘simulated’ growth suppression can also be achieved by modulating the nitrogen content of the nutrient supply. The latter would inevitably induce a transient period, during which the cells transition from growth phase to a lipid accumulation phase, thus adding an extra complication to the single-cell investigation.

Two CHX concentrations were employed. The first was below the minimum inhibitory concentration (“sub-MIC”) at 16 μg/ml [32], where cells still grow, albeit at longer lag-phases and slower growth-rates as determined in liquid batch cultures (S1 Fig). The second was the minimum inhibitory concentration (“MIC”) at 33 μg/ml, where the cells exhibit no/minimal growth, but rather a prolonged stationary phase (S1 Fig). For both CHX concentrations, the proportion of cells exhibiting net lipid degradation by the 140^th^ min decreased substantially, to approximately 25% (45% in the absence of CHX–Fig 4A). Moreover, the sub-MIC concentration lead to a median 30% lipid content increase (Fig 4D), while the MIC concentration to a 60% median lipid content increase (Fig 4G) after 140 min of immobilization.

**Fig 5:**
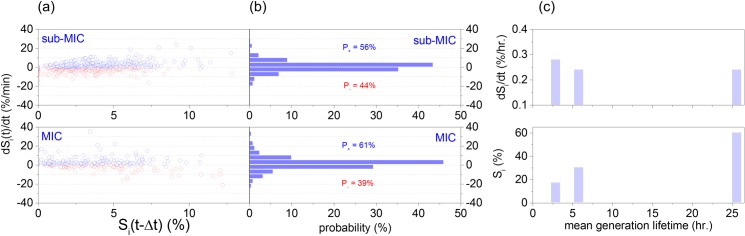
Similar to Fig 2, a scatter plot of the lipid flux (dS_i_/dt) as a function of the total lipid content (S_i_), and the associated probability histogram of the lipid flux for the sub-MIC (a) and for the MIC (b) concentration of CHX. (c) The dependence of the lipid content S_i_ (in % per cell size by the 140^th^ min. of observation) and the instantaneous lipid flux dS_i_/dt (%/hr) on the mean generation lifetime, controlled by different levels of CHX.

Regarding the mechanistics of lipid accumulation, both levels of protein translation inhibition (sub-MIC and MIC) induced lipid accumulation primarily through the number of cytosolic LDs rather than their area, as shown in Fig 4E and 4F, Fig 4H and 4I and S2 Fig. In addition, Fig 5A indicates that both lipid biogenesis and degradation are independent of the cytosolic lipid content and occur at comparable fluxes. Furthermore, complete inhibition of protein translation was accompanied by an enhanced probability of positive rather than negative lipid-flux observations, as illustrated in Fig 5B. Specifically, while either conditions of zero or sub-MIC concentrations of CHX resulted in equally distributed positive and flux observations, the MIC of CHX exhibited 22% higher positive flux observations than negative. The preceding analysis evidences that increasing the inhibition of protein translation and growth induce increasing cytosolic lipid levels (Fig 5C); nevertheless, this increase does not correlate with an increase in the instantaneous lipid flux (Fig 5C), but rather with a decreased probability of lipid degradation (Fig 5B).

## Discussion

Gene expression stochasticity imposes protein copy-number variability between individual cells in isogenic cultures. This form of phenotypic diversity was discovered decades ago [33], and can only be unmasked at the single-cell level [34]. Following substantial interest in the stochasticity of gene expression [35–40], infection [41], and cell growth [42–44], such stochastic fluctuations have been recently applied to the discovery of many unknown aspects of gene regulation [22, 23]. Here, we similarly employed the recently discovered innate fluctuations of lipid accumulation [17] (Fig 1C) to access the mechanistics of lipid regulation, including both *de novo* biogenesis and degradation.

Through single-cell microfluidics and optical microscopy, we determined the positive and negative instantaneous lipid flux for individual cells (Fig 2A). Under microfluidic immobilization and steady-state flow of rich medium, we observed that lipid accumulation and degradation occur at comparable fluxes. These fluxes also appear independent to the cytosolic concentration of product as shown in Fig 2B. Both the comparable accumulation and degradation fluxes and their independence to the cytosolic product concentration suggest that lipid accumulation and degradation undergo similar levels of regulation in rich medium as probed by way of example here for *Y*. *lipolytica*.

Two prominent mechanistic models characterize lipid accumulation. The first suggests that LDs originate in the endoplasmic reticulum (ER) [3, 4, 14]; the second indicates that LDs can also grow by size [16]. By performing longitudinal fluctuation analysis, we conclude that both mechanistic pathways are possible in *Y*. *lipolytica*. This is expected given that both glycerol-3-phospate acyltransferase isoenzyme (GPA) and diacylglycerol acyltransferase (DGA2) are conserved in *Y*. *lipolytica* [13]. However, lipid accumulation is more likely to occur through the number of LDs rather than their size, as evidenced by the correlation analysis presented in Fig 3D and Fig 3C. This evidences that lipid biogenesis at the ER is more probable than increases in the LD size. In regards to lipid degradation, Fig 3C indicates that it similarly occurs through decreases in the number of cytosolic LDs, rather than their size. As a result, we anticipate that the most dominant phenotype in wild-type *Y*. *lipolytica* to be the *small-but-more* LDs (Fig 3A).

The abovementioned behavior was confirmed for both rich medium and protein translation inhibition conditions (Fig 4E and 4F and Fig 4H–4L and S2 Fig). Additionally, the introduction of a protein translation inhibitor (CHX) at sub-MIC and MIC concentrations induced a 2- and 4-fold higher lipid content in comparison to the absence of protein translation inhibition, and correspond to a 2- and 8-fold decrease in growth-rates (Fig 5C) [45]. Finally, we observed that this increase in lipid accumulation was accompanied by a 20% higher probability of lipid accumulation rather than degradation (Fig 5B). While this observation cannot be immediately generalized to nitrogen limiting conditions, it is in agreement with reports on the transcriptional downregulation of β-oxidation in the oleaginous yeast *Rhodosporidium toruloides* [46] and *Y*. *lipolytica* grown in nitrogen limiting media [20].

To our knowledge, this is the first global investigation of the mechanistic regulation of lipid accumulation and degradation. Such mechanistic insight is critical towards engineering enhanced lipogenesis microorganisms as well as improved treatment of obesity and diabetes [47, 48]. In addition, the presented methodology expands upon single-cell investigations of gene regulatory networks to bioprocessing and metabolism, and therefore will provide an experimental platform towards similar investigations.

## Materials and Methods

### Microfluidics

To investigate the mechanistics of neutral lipid accumulation, we employed a single-cell assay, as previously described [17]. Briefly, microfluidics were used to isolate and immobilize individual cells in a microarray format under continuous laminar microflows, following the design by Tan et al. [49, 50]. Following cell immobilization (Fig 1B), we employed 1 μL/min flow rates to both sustain cell trapping and enable rapid replenishment of the environment, thereby giving rise to a quasi-time invariant extracellular environment. The latter is a crucial parameter in our time-dependent metabolic investigation, due to the highly dynamic character of the metabolome, which is known to react with the environment within very short time scales, in the order of a few seconds or less [26]. For this reason, the employed flow rates that are higher than typically employed in suspension cultures, but comparable to the flow rates typically employed in cell growth microfluidic chemostats [51], and substantially lower than those employed in flow cytometry. Under such conditions, we anticipate some minor loss of small molecular weight material from the cell, but given the low mechanical perturbation induced by these flow rates (see [17] for a more detailed discussion on yeast biomechanics under such flow-rates), cells are expected to be able to reach equilibrium under such conditions.

### Cell and culture conditions

The immobilized cells were collected from a batch culture grown for 24h in rich YPD medium. Immobilized cells that exhibited no lipid content, or were actively dividing were not taken into consideration. Under microfluidic immobilization conditions, the neutral lipid content of the cells and the cell size was monitored every 20 min for approximately 140 min.

### Microscopy

Cell size was monitored by bright field microscopy, while the lipid content was determined using Vesicle Photonics [52], coupled to a spin-disk confocal microscope (Fig 1A). For the latter, we employed the lipophilic fluorescent dye Bodipy (BODIPY® 493/503 (4,4-Difluoro-1,3,5,7,8-Pentamethyl-4-Bora-3a,4a-Diaza-*s*-Indacene, Molecular Probes), due to its enhanced specificity to LDs [53]. Cells were stained prior to microfluidic immobilization (250 ng/ml). To prolong the lipid content imaging, the Bodipy stain was included in the medium supplied during microfluidic immobilization (100 ng/ml). Under such conditions, a constant bodipy dye uptake was attained, as evidenced by the monotonic intracellular fluorescence increase shown in S3 Fig, contrary to the lipid content fluctuations shown in the same figure. It is worth adding, that similar dye uptake kinetics are observed in fixed cells; however, lipid content fluctuations are absent in this case, as illustrated in S3 Fig.

More detailed information on the employed cell strain, the procedures of cell growth, microfluidic fabrication, microscopy, image processing, and data analysis are included in the S1 Document.

## Supporting Information

S1 Document Supplementary Information Section. This section describes the derivation of Eq 2, as well as further details the materials and methods information employed in this work (including imaging, image/data analysis, sample preparation, and microfluidics fabrication).
(PDF)

S1 Fig *Yarrowia lipolytica* growth under protein translation inhibition conditions. Growth curves measured for Po1g in YPD medium at three concentrations of cycloheximide (CHX): 0 μg/ml (unperturbed), 16 μg/ml (sum-minimum inhibotory concentration–sub MIC), and 33 μg/ml (minimum inhibotory concentration–MIC). The measurements were performed in a 96-well plate using a Bioscreen C Pro instruement; each measurement represents an average of four independent wells at a 100x dilution.
(PDF)

S2 Fig Lipid flux dependence on the LD size and copy-number under protein translation inhibition conditions. The dependence the lipid flux (dS_i_/dt) on [α_i_ ∙ dN_i_/dt] (red circles) and [N_i_ ∙ dα_i_/dt] (blue circles) for the Po1g strain under sub-minimum inhibitory concentrations (sub-MIC–left) and minimum inhibitory concentrations (MIC–right). Each data point denotes a single observation per unit time per cell, and the solid lines illustrate linear fits; insets include the Spearman correlation coefficient (ρ).
(PDF)

S3 Fig LD fluorescence staining and dye uptake analysis for live and fixed cells. (a) A graph illustrating the dynamics of the intracellular fluorescence intensity (“fluorescence”) of the Bodipy dye, the total lipid content (S_i_ in %), and the ratio of the fluorescence intensity of the propidium iodide (PI) dye (“PI ratio”). The latter denotes the ratio of the intracellular PI fluorescence over the extracellular fluorescence, which is less than 1 for live cells. (b) The same graph for fixed cells (PI ratio >1), where the lipid content fluctuations are reduced, despite the similar dye uptake kinetics.
(PDF)
