# Supplementary material for: Exploiting Bioprocessing Fluctuations to Elicit the Mechanistics of De Novo Lipogenesis in Yarrowia lipolytica
Source: PLoS One. 2017 Jan 4;12(1):e0168889. doi: 10.1371/journal.pone.0168889 (PMC5215641; doi:10.1371/journal.pone.0168889)
Supplement: S1 Document — This section describes the derivation of Eq 2, as well as further details the materials and methods information employed in this work (including imaging, image/data analysis, sample preparation, and microfluidics fabrication). (PDF) [file pone.0168889.s001.pdf]

Supplementary Information Section

Exploiting Bioprocessing Fluctuations to Elicit the Mechanistics of *de novo* Lipogenesis in *Yarrowia lipolytica*

A. E. Vasdekis[1,2,*], A. M. Silverman[3], G. Stephanopoulos[3]

[1] Department of Physics, University of Idaho, Moscow, ID, 83844, USA.

[2] Molecular Sciences Laboratory, Pacific Northwest National Laboratory, Richland, WA, 99354, USA.

[3] Department of Chemical Engineering, Massachusetts Institute of Technology, Cambridge, MA 02139, USA.

I. Lipid Accumulation Flux

Generally two broad cascade elements are involved in lipid biogenesis, namely carbon internalization and intracellular metabolic reactions (highlighted in green in the block diagram below). Intracellular carbon is denoted as C_i, neutral lipid product as S_i and the related enzyme manifolds for lipid synthesis and degradation as E^+ and E^- respectively (with k^+ and k^- being their reaction rates).

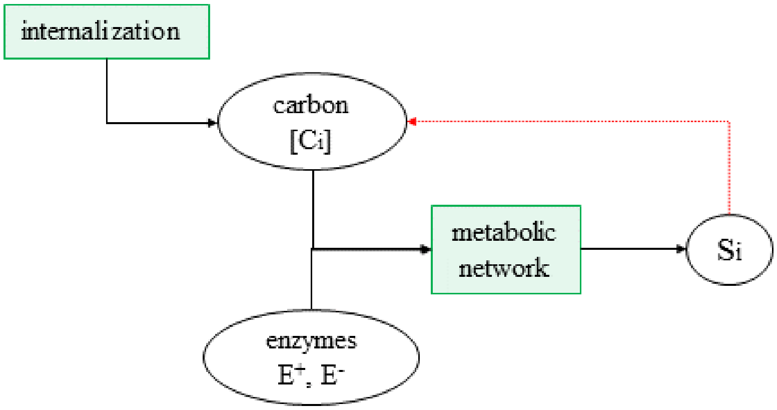

Under the assumption that intracellular carbon is at equilibrium with the quasi-time-invariant extracellular supply (i.e. C_i: constant, while S_i, E^+, E^-, k^+ and k^- being time dependent), lipid biogenesis may be further simplified as a single enzymic step reaction, where synthesis and degradation enzyme manifolds are characterized by the k^+ and k^- reaction rates [1]:

$$[C_i],\ E^+,\ k^+ \ \rightleftharpoons\ S_i\,, E^-,\ k^-$$

Using the law of mass action, this reaction scheme can be re-written as:

$$S_i(t) = S_i(t-\Delta t) + S_{new}(\Delta t) - S_{degraded}(\Delta t)$$

$$\Rightarrow \quad S_i(t) - S_i(t-\Delta t) = S_{new}(\Delta t) - S_{degraded}(\Delta t)$$

$$\xRightarrow{C_i:\text{constant}} S_i(t) - S_i(t-\Delta t) = [\kappa^+(t)\cdot E^+(t)\cdot(S_i(t-\Delta t)+C_i)] - \kappa^-(t)\cdot E^-(t)\cdot S_i(t-\Delta t)$$

$$\Rightarrow \quad dS_i(t) = \kappa^+(t)\cdot E^+(t)\cdot C_i + [\kappa^+(t)\cdot E^+(t) + \kappa^-(t)\cdot E^-(t)]\cdot S_i(t-\Delta t)$$

$$\Rightarrow \frac{dS_i(t)}{dt} = \frac{\kappa^+(t) \cdot E^+(t)}{dt} \cdot C_i + \frac{\kappa^+(t) \cdot E^+(t) + \kappa^-(t) \cdot E^-(t)}{dt} \cdot S_i(t - \Delta t)$$

This differential equation represents the abovementioned reaction scheme, with blue denoting lipid synthesis and red lipid degradation. This rate equation indicates that temporal fluctuations of the lipid bioprocessing rate (dS_i/dt) generally emanate from changes in the concentration (E) and reaction rates (κ) of related enzyme manifolds, as well as the instantaneous product concentration (S_i).

II. Materials and Methods

Strains and Culture Conditions: All experiments were performed using the oleaginous yeast *Yarrowia lipolytica*. Specifically, we employed the Po1g strain (Yeastern Biotech Company, Taipei, Taiwan), the details and extensive characterization of which have been previously presented in reference [2]. The cells were grown in YPD medium, with glucose as the carbon source (10g/L Yeast Extract – Difco Laboratories, 20g/L Bacto-Peptone – Difco and 20g/L glucose – Sigma Aldrich). Where indicated, cycloheximide (CHX - Sigma Aldrich) was added to the medium flowing in the microfluidics following immobilization at concentrations of 16 μg/ml and 33 μg/ml [3, 4]. CHX was dissolved in DMSO (Molecular Probes) at a 330 mg/ml concentration, and divided in 20 μL aliquots for storage at -20°C until further use. DMSO was chosen for dissolving CHX due to the high solubility of the lipid stain in the same solvent (see below). The total DMSO concentration in the medium was kept at 0.02%, well below the 1% concentration rendered as having no impact on yeast physiology [5, 6]. In comparing the lipogenesis effects of protein translation inhibition, care was taken so that the total DMSO concentration in the medium was the same in all cases (0.02%).

Cell Growth and Sampling: Cells were grown at room temperature (regulated at 25°C±0.5^{o}C) under orbital shaking conditions (180 rpm). For this, cells were transferred to sterile Falcon tubes containing 5 ml of YPD medium at a 50x dilution. In all experiments, the cells were harvested during early stationary phase at 24h (OD_{600} ~ 1.5 – **S1 Fig**). At

this time-point, the cells exhibit a higher lipid content in comparison to earlier growth stages as we previously reported [7].

Sample Preparation: For single-cell analysis in microfluidics, a 1.5 ml cell suspension (1x dilution) was collected following 24h of continuous growth. To fluorescently label the lipid droplets, a bodipy dye (BODIPY® 493/503 (4,4-Difluoro-1,3,5,7,8-Pentamethyl-4-Bora-3a,4a-Diaza-*s*-Indacene – Molecular Probes) solution in DMSO (Molecular Probes) was added in YPD medium at a concentration of 250 ng/ml, followed by a 4 hour long incubation. Subsequently, cells were transferred to a gastight syringe at a 10x dilution in YPD medium containing a lower concentration of the bodipy dye (100 ng/ml) and loaded to the microfluidic traps. Following immobilization, the same solution (YPD-Bodipy concentration 100 ng/ml, 0.02% DMSO) was continuously supplied through a second inlet at a rate of 1 μL/min to enable stable immobilization. A 40 ng/ml propidium iodide solution in water (Sigma Aldrich) was included in the medium to detect cell apoptosis. Under such conditions, a constant fluorescent dye uptake was attained (see **S3 Fig**), contrary to the fluctuating lipid content of individual cells. This evidences that the lipid content variations emanate from inherent bioprocessing fluctuations, rather than unstable staining conditions. To a similar end, such lipid content fluctuations were not observed in fixed cells, despite similar dye uptake kinetics (see **S3 Fig**). On average ~ 80 individual cells per growth condition were studied.

Microfluidics: The microfluidics were fabricated with soft lithography by molding polydimethilsiloxane (PDMS, Sylgrad 184, Dow Corning) and bonding on a glass coverslip [8]. Briefly, a 10:1 monomer to catalyst ratio was manually mixed and degassed for 2h in a vacuum desiccator. The mixture was subsequently poured over a mask (SU8 photoresist on a silicon substrate) and baked for 2 hours at 70°C, followed by cutting to appropriate dimensions, plasma bonding to glass coverslips and overnight baking at 70°C. Two inlets enabled cell loading and continuous medium delivery, and one outlet enabled waste collection. Medium and cell flow were enabled by two syringe pumps (Harvard Apparatus) and Tygon tubing (inner diameter 0.02''). The cell trapping design was based on the Tan and Takeuchi axial percolation filtration method [9, 10]. 16 trapping sites were arrayed connected to a cell-loading and a medium-delivering channel (50 μm

wide – 10 μm thick). The traps comprised of a dwell chamber and a narrow indentation with approximate widths 6 μm and 2.5 μm respectively. On average, 7-9 cells were trapped per experiment, with an approximately a 50% trapping efficiency.

Imaging: Vesicle photonics through confocal fluorescent microscopy was employed for visualizing the stained LDs [11], using an inverted microscope (Leica DMI6000) integrated with a spinning disk confocal apparatus (Yokogawa, CSU10). A two wavelength laser illumination (488 nm for lipids and 532 nm for viability and apoptosis detection) as well as bright-field imaging (for cell size) were employed. Images were acquired through a 100x oil objective (100x/1.4 NA) on a CCD array (Photometrics CoolSNAP HQ2, pixel size 6.45 μm x 6.45 μm) with typical acquisition settings of a 300 msec integration time, ~10 mW optical excitation on the sample for both lines and a z-scanning step of ~250 nm.

Each cell required approximately 1 min for optical sampling at all three spectral channels, which imposed an upper temporal resolution limit. Under such imaging and staining conditions, the smallest detectable lipid droplet was of an area equal to 0.09 μm^2. This limit was set by requiring a signal to noise > 2. In the context of spatial resolution (~200 nm according to the manufacturer), a single dark pixel criterion was used to distinguish two lipid droplets.

Due to the lower axial resolution (i.e. along the z-axis) of the microscope than the lateral one (xy-plane), we quantified the size of the lipid droplets using the maximum intensity projection analysis [7, 12]. Following acquisition, the images were stored for processing and analysis using ImageJ. For processing, the 3D confocal images were converted to 2D through the maximum intensity projection analysis, followed by convolution with a Gaussian (radius of decay = standard deviation – σ) to attenuate its low spatial-frequency components. Subsequently a bandpass filter was applied to the images (filter size equal to maximum feature size), followed by subtracting the filtered image from the original one (ImageJ). The lipid droplets were then identified by applying thresholding based on histogram entropy [13]. In this way, the number and area of individual lipid droplets was determined and normalized over the cell area as determined by bright field imaging.

Data Analysis: Due to the presence of outliers in our observations, robust statistics were employed in data analysis (see for example [14]). To this end, a custom script was written

in Matlab for performing the statistical analysis for the longitudinal fluctuations and noise determination. Linear fits and correlation analyses were performed in Origin Pro (OriginLab, 64Bit, 2015).

Supplementary References

1. Frenzen CL, Maini PK. Enzyme Kinetics for a 2-step Enzymic Reaction with Comparable Initial Enzyme-Substrate Ratios. Journal of Mathematical Biology. 1988;26(6):689-703. PubMed PMID: WOS:A1988R352400006.

2. Tai M, Stephanopoulos G. Engineering the push and pull of lipid biosynthesis in oleaginous yeast Yarrowia lipolytica for biofuel production. Metabolic Engineering. 2013;15:1-9. doi: 10.1016/j.ymben.2012.08.007. PubMed PMID: WOS:000313882500001.

3. Belle A, Tanay A, Bitincka L, Shamir R, O'Shea EK. Quantification of protein half-lives in the budding yeast proteome. Proceedings of the National Academy of Sciences of the United States of America. 2006;103(35):13004-9. doi: 10.1073/pnas.0605420103. PubMed PMID: WOS:000240380800013.

4. Schneider-Poetsch T, Ju J, Eyler DE, Dang Y, Bhat S, Merrick WC, et al. Inhibition of eukaryotic translation elongation by cycloheximide and lactimidomycin. Nature Chemical Biology. 2010;6(3):209-17. doi: 10.1038/nchembio.304. PubMed PMID: WOS:000274565100013.

5. Murata Y, Watanabe T, Sato M, Momose Y, Nakahara T, Oka S, et al. Dimethyl sulfoxide exposure facilitates phospholipid biosynthesis and cellular membrane proliferation in yeast cells. Journal of Biological Chemistry. 2003;278(35):33185-93. doi: 10.1074/jbc.M300450200. PubMed PMID: WOS:000184901800085.

6. Sadowska-Bartosz I, Paczka A, Molon M, Bartosz G. Dimethyl sulfoxide induces oxidative stress in the yeast Saccharomyces cerevisiae. Fems Yeast Research. 2013;13(8):820-30. doi: 10.1111/1567-1364.12091. PubMed PMID: WOS:000326842900010.

7. Vasdekis AE, Silverman AM, Stephanopoulos G. Origins of Cell-to-Cell Bioprocessing Diversity and Implications of the Extracellular Environment Revealed at the Single-Cell Level. Scientific Reports. 2015;5. doi: 10.1038/srep17689. PubMed PMID: WOS:000366283800001.

8. Thorsen T, Maerkl SJ, Quake SR. Microfluidic large-scale integration. Science. 2002;298(5593):580-4. doi: 10.1126/science.1076996. PubMed PMID: WOS:000178634800035.

9. Tan W-H, Takeuchi S. A trap-and-release integrated microfluidic system for dynamic microarray applications. Proceedings of the National Academy of Sciences of the United States of America. 2007;104(4):1146-51. doi: 10.1073/pnas.0606625104. PubMed PMID: WOS:000243849900009.

10. Vasdekis AE. Single microbe trap and release in sub-microfluidics. RSC Advances. 2013;3(18):6343-6. doi: 10.1039/c3ra40369f. PubMed PMID: WOS:000317584200021.

11. Vasdekis AE, Scott EA, Roke S, Hubbell JA, Psaltis D. Vesicle Photonics. Annual Review of Materials Research, Vol 43. 2013;43:283-305. doi: 10.1146/annurev-matsci-071312-121724. PubMed PMID: WOS:000323892700012.

12. Chumnanpuen P, Brackmann C, Nandy SK, Chatzipapadopoulos S, Nielsen J, Enejder A. Lipid biosynthesis monitored at the single-cell level in Saccharomyces cerevisiae. Biotechnology Journal. 2012;7(5):594-601. doi: 10.1002/biot.201000386. PubMed PMID: WOS:000303190900009.

13. Sahoo PK, Soltani S, Wong AKC, Chen YC. A survey of thresholding techniques. Computer Vision Graphics and Image Processing. 1988;41(2):233-60. doi: 10.1016/0734-189x(88)90022-9. PubMed PMID: WOS:A1988M058600007.

14. Committee AM. Robust statistics: a method of coping with outliers. AMC Technical Briefs. 2001;6.
