# Supplementary figures and images for: Exploiting Bioprocessing Fluctuations to Elicit the Mechanistics of De Novo Lipogenesis in Yarrowia lipolytica
Source: PLoS One. 2017 Jan 4;12(1):e0168889. doi: 10.1371/journal.pone.0168889 (PMC5215641; doi:10.1371/journal.pone.0168889)

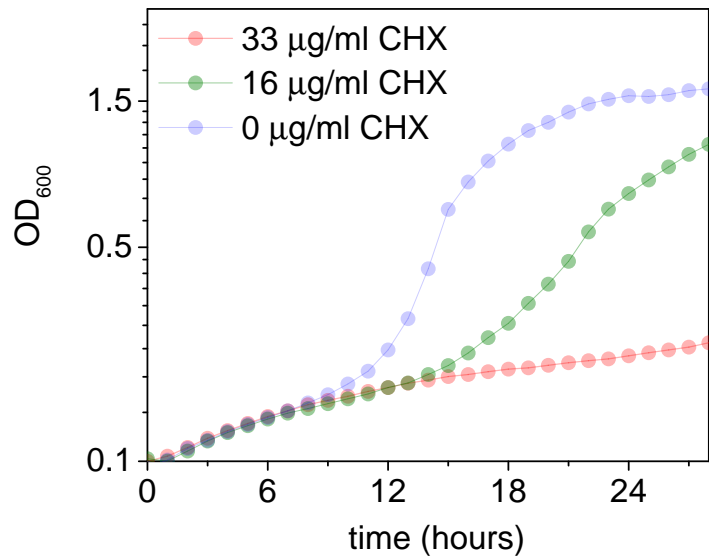

Supplement: S1 Fig — Growth curves measured for Po1g in YPD medium at three concentrations of cycloheximide (CHX): 0 μg/ml (unperturbed), 16 μg/ml (sum-minimum inhibotory concentration–sub MIC), and 33 μg/ml (minimum inhibotory concentration–MIC). The measurements were performed in a 96-well plate using a Bioscreen C Pro instruement; each measurement represents an average of four independent wells at a 100x dilution. (PDF) [file pone.0168889.s002.pdf]

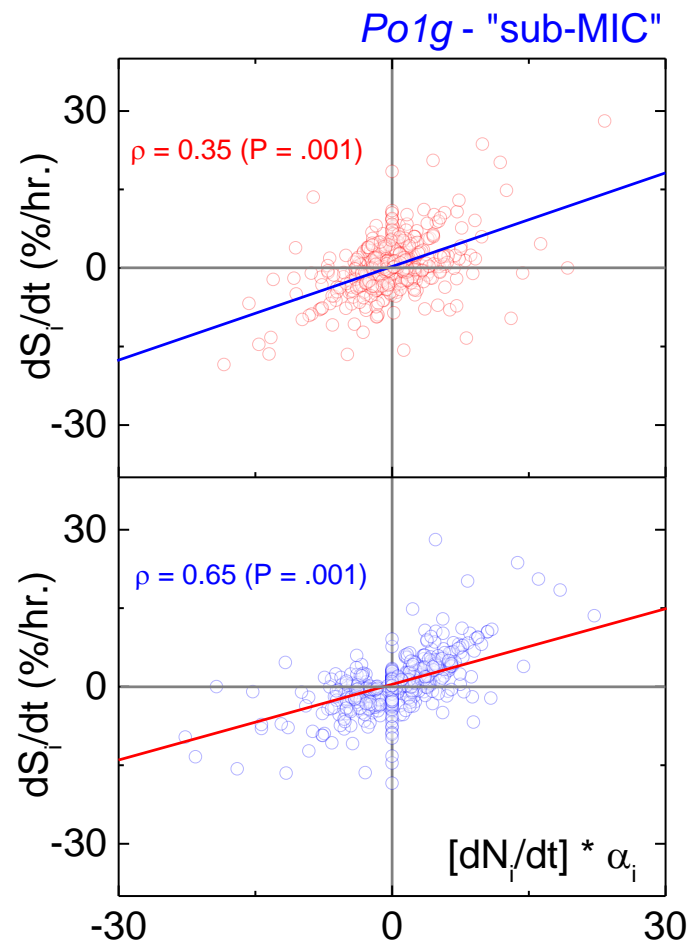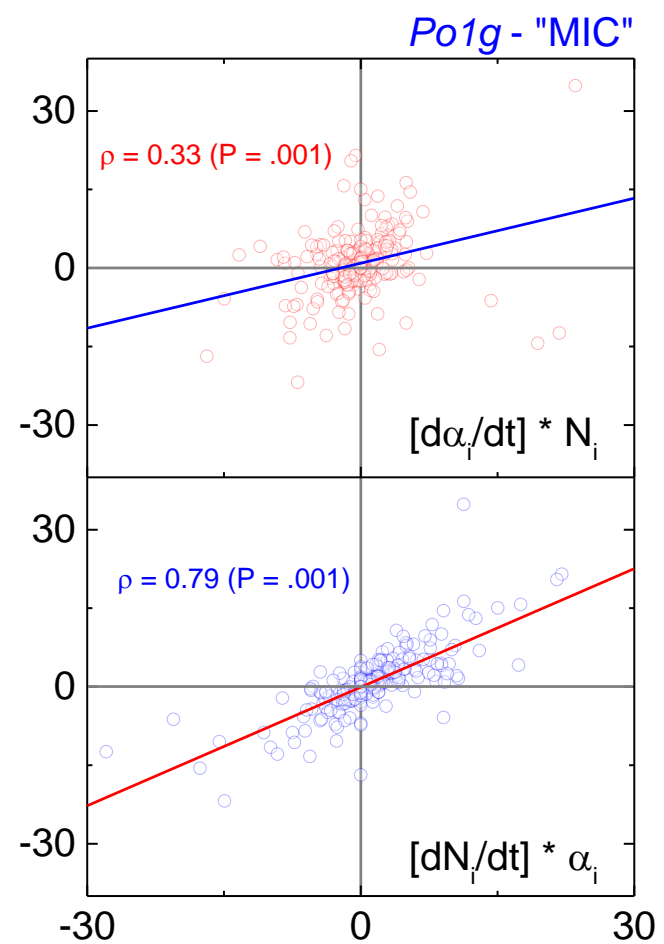

Supplement: S2 Fig — The dependence the lipid flux (dSi/dt) on [αi ∙ dNi/dt] (red circles) and [Ni ∙ dαi/dt] (blue circles) for the Po1g strain under sub-minimum inhibitory concentrations (sub-MIC–left) and minimum inhibitory concentrations (MIC–right). Each data point denotes a single observation per unit time per cell, and the solid lines illustrate linear fits; insets include the Spearman correlation coefficient (ρ). (PDF) [file pone.0168889.s003.pdf]

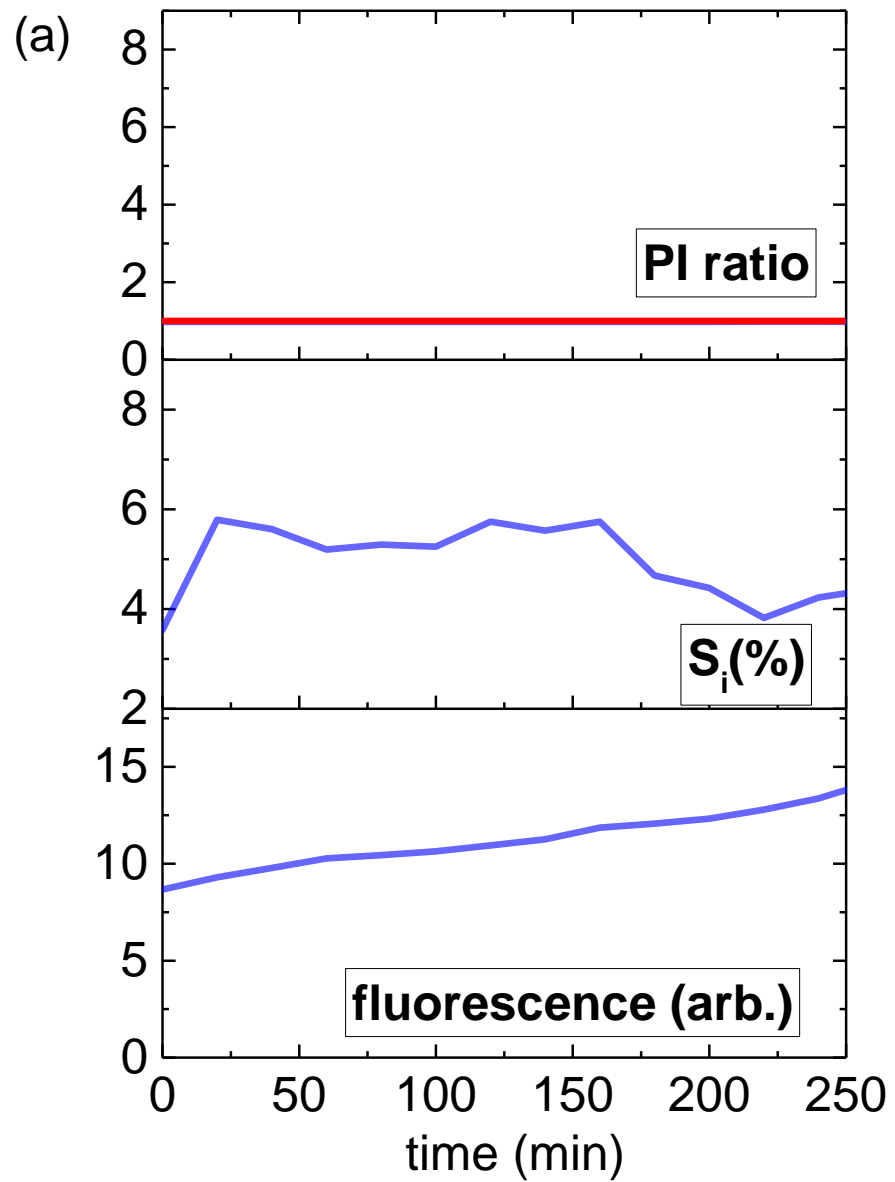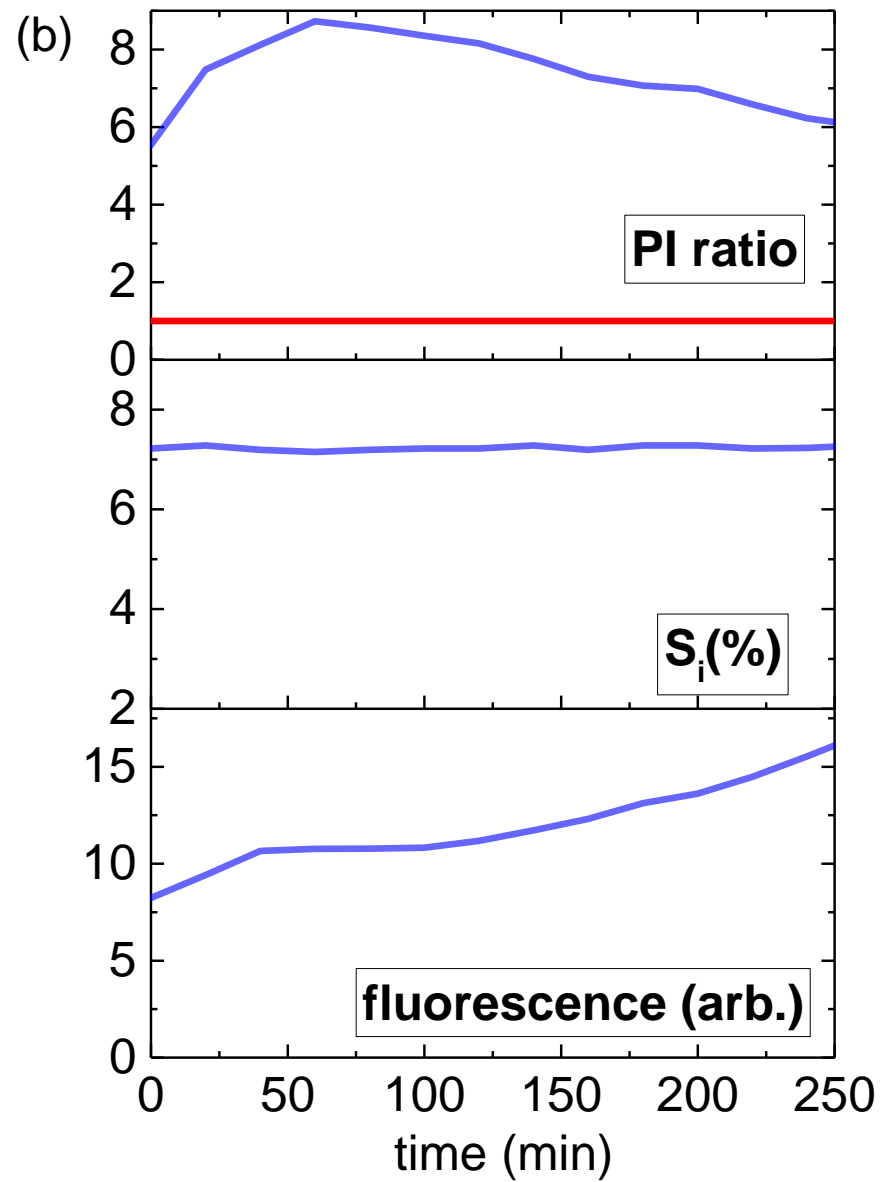

Supplement: S3 Fig — (a) A graph illustrating the dynamics of the intracellular fluorescence intensity (“fluorescence”) of the Bodipy dye, the total lipid content (Si in %), and the ratio of the fluorescence intensity of the propidium iodide (PI) dye (“PI ratio”). The latter denotes the ratio of the intracellular PI fluorescence over the extracellular fluorescence, which is less than 1 for live cells. (b) The same graph for fixed cells (PI ratio >1), where the lipid content fluctuations are reduced, despite the similar dye uptake kinetics. (PDF) [file pone.0168889.s004.pdf]
